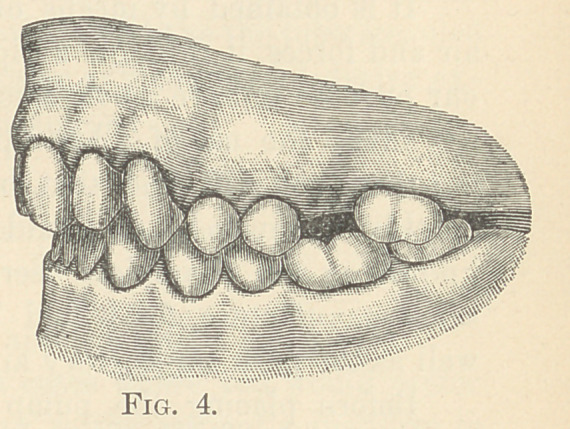# Is It Possible to Jump the Bite?

**Published:** 1900-03

**Authors:** Eugene S. Talbot


### IS IT POSSIBLE TO JUMP THE BITE?

BY EUGENE S. TALBOT, M.D., D.D.S.[1]

UNDER the above title appears in the February (1900) *Dental Cosmos* a paper read before the First District Dental Society of New York by Dr. Rodrigues Ottolengui, which presents some comical features.[2]

"Jumping the bite," he remarks, "means only one thing. It means the movement of the whole jaw so that it bites in a new position, and that position must be forward and not backward. There never has been a bite jumped backward, and there never will be in my opinion.

"My conception of the etiology of these cases is that the lower jaw is behind its proper position. That is, regardless of the deformity in the upper jaw, the lower retreats to a degree which would cause an asymmetry of the external features, even were the upper jaw normal. This being true, it is manifest that a reduction of the upper prominence to a close occlusion with the lower, leaving the latter in its retreated pose, would really be carrying the upper incisors back beyond the line required by beauty, and the retreat of the chin would be as marked a disfigurement as ever. On the contrary, if the upper jaw be reduced to normal only, and the lower be brought forward so that the teeth and lips meet comfortably, the best cosmetic result will be attained. This is to be accomplished by jumping the bite and by no other way.

"In the case which I present to-night (Fig. 1), it was evident at the outset that the upper jaw should be widened, the protrusion reduced, and the bite jumped, as the only means of restoring both the internal and external symmetry. The widening accomplished, the reduction was effected with a gold spring wire, after the method suggested by Dr. Jackson. A plate was made of iridio-platinum fitted with clasps about the molars, to which were attached the ends of a wire which came around the front of the arch. This was made of clasp gold, a loop occurring in the region of each cuspid. These loops are closed from time to time, and afford the tension to the front part of the wire which gradually reduces the arch. This method was adopted because the spaces between the teeth offered opportunity

---

[1] Fellow of the Chicago Academy of Medicine.

[2] I have copied such parts as are of interest at the present time. The entire article with the discussion will well repay perusal.

for the reduction of the antero-protrusion with much straining upon the anchorage, the plate, however, being relied upon to reduce this strain by giving a bearing against the vault. The correction was readily accomplished and the retainer was the same plate, with a restraining band around the arch, the plate being fitted with the inclined plane which was intended to jump the bite. The models and examination of the mouth disclose that the end was accomplished.

"There is one feature of this case to which special allusion should be made. In the discussion of Dr. Talbot's paper, he advanced the theory that an attempt to move the lower jaw forward would open the bite in the bicuspid region, and he asked those present to make the attempt and see whether a knife-blade could not be passed between the teeth. Dr. Talbot's mistake in that was that he overlooked the fact that before jumping the bite we widen the upper jaw, thus affording opportunity to move the lower jaw forward without opening the bite. Usually this can be accomplished, but it must be confessed that it is not always so, that there are cases where Dr. Talbot's theory holds, even though the upper jaw be widened. This case which I present, was, in a limited degree, of this class. The pose of the molars was such, that in sliding the lower jaw forward the bite opened slightly in the bicuspid region.

"In closing, I desire to make plain my purpose to-night. Dr. Talbot suggested that a case of jumping the bite should be submitted to a committee of three who should report as to the result. It is not easy to find a patient who would consent to appear before a body of scientific men, and I did not imagine until quite recently that such a request would be granted. Consequently the case presented to-night was completed before I thought of showing the patient personally. However, I assure you the models of the original case are accurate, and ask that in the interest of truth in science, those who have examined the mouth and models, will, during the discussion, express an opinion as to the result, remembering that it is about one year and a half since the appliance for jumping the bite was introduced."

This article appeared to me at first merely a practical joke. Turning to the discussion, I was astonished to see that it was seriously considered by the members of the First District Dental Society. The questions naturally occurred: Does Dr. Ottolengui mean what he says? Does he really think he has jumped the bite? Does he really think such an operation possible? Does he believe Dr. Kingsley did it? To answer these questions one must follow Dr. Ottolengui in his paper.

I understand "jumping the bite" to be (exactly as expressed by both Dr. Kingsley and Dr. Ottolengui) a bodily forward movement of the lower jaw at the glenoid cavity, the width of a bicuspid tooth. The "backward" movement of the condyles in the glenoid cavity was added by me because it seemed just as reasonable for the patient to move the jaw backward as forward. When Dr. Ottolengui says that "my conception of the etiology of these cases is that the lower jaw is behind its proper position. That is, regardless of the deformity in the upper jaw, the lower retreats to a degree which would cause an asymmetry of the external features even were the upper jaw normal," he does not seem to understand the etiologic factors of the tissues under discussion. This being the case, he could easily become confused as to the method of treatment and the results obtained.

The tissues of the body when not normal are either excessively developed or arrested from unstable nervous control. In this case there is arrest of development of the lower jaw. The lower jaw does not "retreat." The articulation at the glenoid cavity is normal. The relation of the lower jaw to the upper is, therefore, in this particular case normal. The correction of this arrest of development, as the doctor says, "is to be accomplished by jumping the bite and by no other way." The question arises, Did he jump the bite?

It is wholly unnecessary to submit the case to a committee of three, since the models before and after the operation said to be "accurate" are here illustrated. A study of the illustrations is all that is needed to demonstrate that the doctor did not "jump the bite." By glancing at the two models, the single molar upon the upper jaw stands in precisely the same position in both models against the lower molars. If the bite had been jumped, the two second molars would have stood one upon the other. In other words, the lower second molar would have moved forward the width of a bicuspid tooth, which would have brought the two second molars together. The lower incisors would stand in front of the lateral, instead of behind as shown in Fig. 1. What has taken place in correcting this deformity? The upper incisors, cuspids, and bicuspids have been carried backward the width of a bicuspid tooth. The space where the superior first permanent molar has been extracted has diminished in width. This has been brought about by the second bicuspid standing behind the second bicuspid upon the lower jaw instead of in front of it. The first bicuspid and cuspid have also dropped backward the width of one tooth. This has given room for the incisors and alveolar process to be carried back into a normal position. It is apparent to everyone that the lower jaw could not be carried forward the width of a bicuspid, and the upper incisors and alveolar process carried backward. Anyone can see that the incisors and alveolar process have been carried backward. Only one operation has been accomplished. It is easy enough to see which of the two movements has taken place. The location of the bicuspids before and after indicate this; the location of the incisors and anterior alveolar process before and after settles that question. It will be seen that just the opposite was accomplished from what the doctor intended. The upper teeth were moved backward instead of the lower jaw being carried bodily forward. He says, "the models (Fig. 4) and examination of the mouth discloses

Fig. 1.

Fig. 4.

that the end was accomplished." That is, he was satisfied with the appearance of the face and jaws. This shows that his judgment was not only faulty as to etiology, but also to the proper method of reducing the deformity to improve the appearance of the face. In this case the results are all that could be expected.

The joke appears to be not only upon Dr. Ottolengui, but also upon the members of the First District Dental Society, in not recognizing what had been accomplished. I have tried in three cases since 1892 to "jump the bite," but have found that in each case, the patient's jaws became so tired at the end of three or four days that it was necessary to give up this method of treatment. I therefore reiterate what I said in my paper read before the New York State Dental Society in 1892, "I have never been able to 'jump the bite,' although I have tried it in a number of cases. I do not believe anyone else has been able to accomplish it, nor do I believe that such a thing is possible."[1]

[1] "Dental Cosmos," 1892, page 791.

## Figures and Tables

**Fig. 1. f1:**
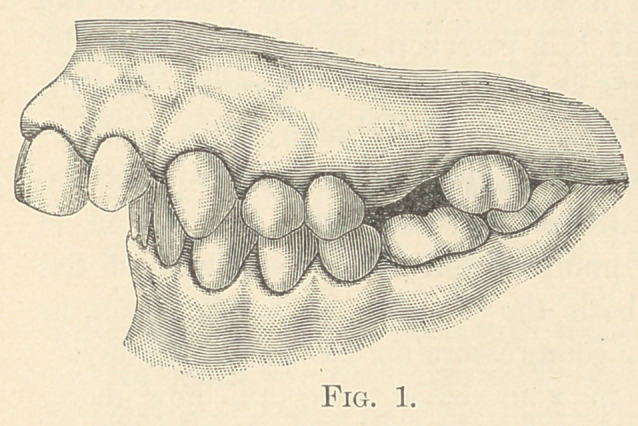


**Fig. 4. f2:**